# Verifications

**Published:** 1887-05

**Authors:** Edward Fornias

**Affiliations:** Philadelphia


### VERIFICATIONS.

EDWARD FORNIAS, M. D., PHILADELPHIA.

### SULPHUR.

In 1877, while living in Harrisburg, Pa., I was consulted by the late Dr. Freese for a case of *intermittent fever* of long duration and repeated attacks, which had before this been overpowered by large doses of *Quinine*, with much detriment to an already broken-down constitution.

The patient was a man of forty-five years of age, and received from Dr. Freese (who, I must say, was a good prescriber) *Ars.* and *Ipecac*, the latter remedy giving the best results, but unable to eradicate the trouble. At a renewed attack of chills I was invited, by the above doctor, to see the patient. I found him very *weak and emaciated*, and complaining of *violent burning in the palms of the hands and the soles of the feet*, which induced me to place my hand on the top of his head, and there I met with a well-known symptom, which I have frequently verified, and which I think is a common concomitant in uterine disorders, namely, *burning heat on the vertex*. This, together with the marked *venosity* exhibited by his hands, was enough indication to suggest to me *Sulphur*, which in the thirtieth D. was given with the best results, only three doses being sufficient to cure the patient radically.

For over a year I followed this case, and during that time the chills never returned.

Now, who would, in view of such results, dare to wipe out these symptoms from our materia medica?

### SILICEA AND FLUORIC ACID.

A cigarmaker, thirty-seven years of age, tired of being probed and tormented by an old-school physician, who had treated him for some time, applied finally to me, stating that when convalescing from a severe attack of *typhoid fever, a tumor appeared in one of the right intercostal spaces*, which, after being frequently poulticed, broke open and commenced to discharge, first, *a thick, creamy pus, mixed with some blood but becoming every day thinner and more offensive*. When I first saw him he was *pale* and *weak*, and the discharge, which by its long duration was causing a decided harm to the organism, consisted of *a thin, dirty, fetid ichorous fluid*, and the surrounding *parts were hard, swollen,*

*and bluish.* He was suffering also from *pain in the ribs,* with the peculiarity that while he was working *in a warm room he felt relieved,* but as soon as he would go *out into the cold air* (being winter at the time in which I commenced to treat him) *his sufferings became unbearable.* Of course, *Silicea* $^{30}$ was prescribed, and its good and infallible effects were soon evinced, the abcess healing completely in six weeks, and the patient gaining much in flesh. So the improvement of the general health continued until the following summer, when, by excesses in drinking, late hours, etc., another abscess was formed, an inch below the old one, which took nearly the same course. *Silicea* in the third, thirtieth, and two hundredth was given again, with little improvement. Studying the case thoroughly, I then found that, contrary to the previous time, my patient felt *much better while working in a cool room, and great deal worse when going out in heat of the sun.* This time *Fluoric acid* $^{30}$ was given, and a complete cure effected, with no return of the trouble ever since. I am sorry to see that our *Materia Medicas* do not give the opposite aggravations of *Silicea* and *Fluoric acid,* upon which Dr. Farrington used to put so much reliance, and which was so clearly confirmed in this case.

### Alumina.

Two interesting cases, which obviously prove that there are hidden treasures in our materia medica worth keeping and protecting, are the following: 1. A rachitic three-year-old child of a lady I had attended in labor was suffering from *atrophy.* According to the mother, the child had been *fed with the bottle* since four months old, suffered great deal from *colic; would not thrive at all, and dentition was very slow.* Up to the time I saw him he *had not yet learned to walk or talk;* his *abdomen* was *distended* and *hard,* with *swollen mesenteric glands;* his *limbs emaciated,* his *knees knobby,* his *head disproportionately large* and *heavy,* the *fontanelles not closed,* his *appetite voracious,* the *thirst constant,* and the *stools pasty, of a clay-like color,* and *attended by much straining.* He received *Calc. carb.,* which he continued to take for a few days in different dilutions, with little improvement, until one day the mother called my attention to an inclination the child had shown of late to pick up and *eat things* he would find on the floor, *such as pieces of paper, earth, etc.,* stating that on that very day he came near choking with a long *string of rag carpet* he was trying to swallow. This, of course, brought *Alumina* to my mind at once, and endeavoring to find "*some*

*more legs for the table to stand upon,"* I learned that the *dry, lustreless hair*, the *absence* of *sweat*, and *the straining at stool*, so well exhibited in the case, together with the *abnormal cravings*, were strong indications to decide in favor of this drug. That the selection was good the results did exceedingly prove, as the child soon became another creature, and is to-day a strong, healthy boy.

2. The other case has brought more patients to my practice than I ever had. It had been given up by the old school and was very much like the above, but worse in every respect. *Fed by the bottle; slow in walking, speaking*, and *cutting teeth; enormously large head, with open fontanelles*, and *bathed in cold sweat; voracious appetite, distended abdomen, glandular involvement, progressive wasting, persistent, watery diarrhœa, etc.*, was the picture of the case. After *Calc.-carb.* and *Sulph.* had done much good and the child was in the way to recovery, a violent fever, with involvement of the brain, set in, which nearly took him to the grave. This alarming condition being mastered, the child went back to his former trouble, when *Alumina* $^{30}$ became the deciding factor in making of him the healthy and strong boy he is to-day.

The indications for *Alumina* were: *Abnormal cravings, liquid stools, with much straining; absence of sweat, dry, lustreless hair*, and *persistent strabismus*, which was left by the fever, and commenced to improve with the other symptoms as soon as this remedy was prescribed.

This little fellow lives in the neighborhood of Third and Spruce Streets, and is at present a complete picture of health.

